# Supplementary figures and images for: Searching for Collective Behavior in a Large Network of Sensory Neurons
Source: PLoS Comput Biol. 2014 Jan 2;10(1):e1003408. doi: 10.1371/journal.pcbi.1003408 (PMC3879139; doi:10.1371/journal.pcbi.1003408)

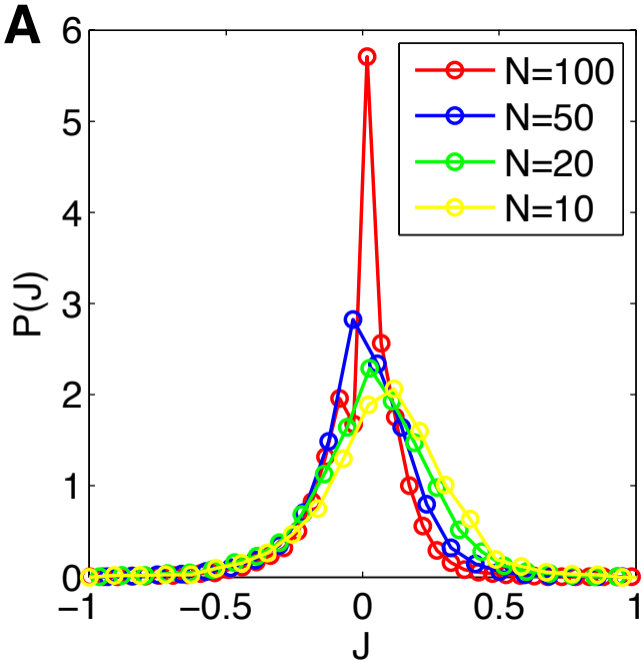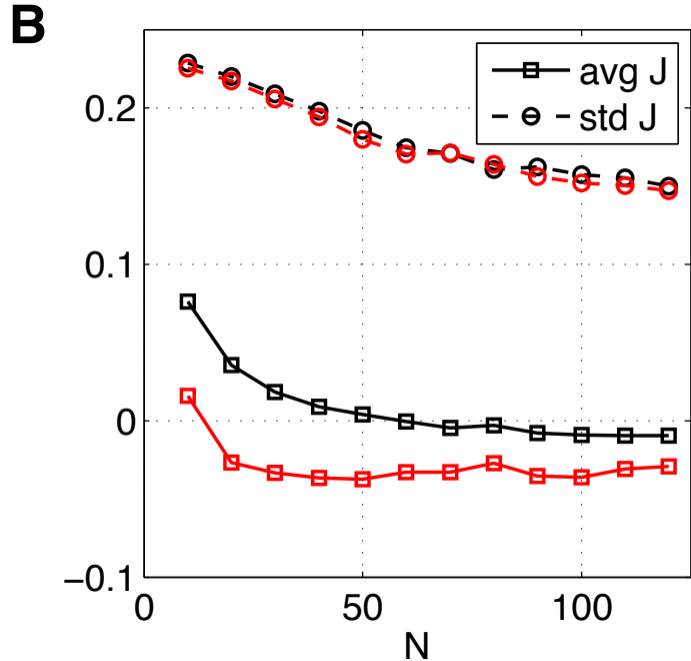

Supplement: Figure S1 — Interactions in the (K-)pairwise model. (A) The distributions of pairwise couplings, , in pairwise models of Eq (19), for different network sizes (N). The distribution is pooled over 30 networks at each N. (B) The mean (solid) and s.d. (dashed) of the distributions in (A) as a function of network size (black); the mean and s.d. of the corresponding distributions for K-pairwise models as a function of network size (red). (PDF) [file pcbi.1003408.s001.pdf]

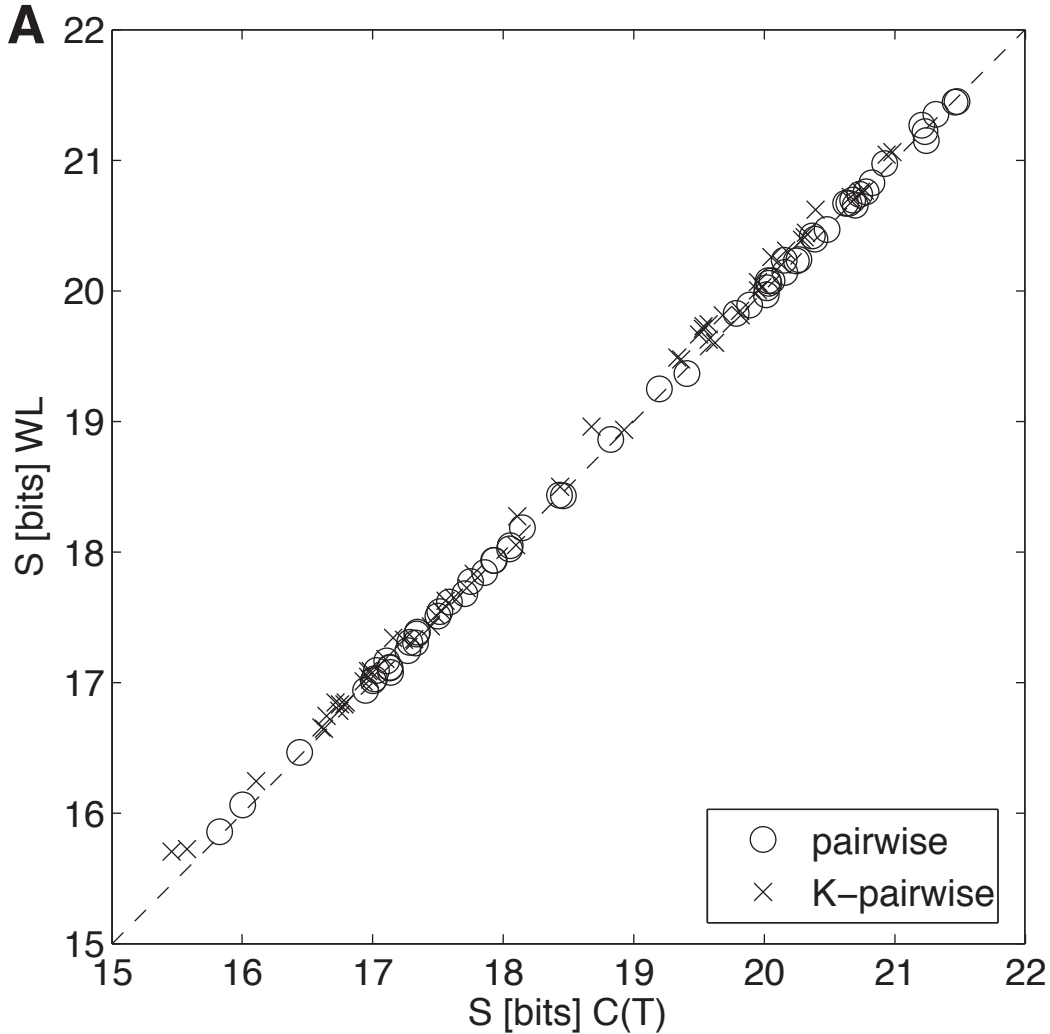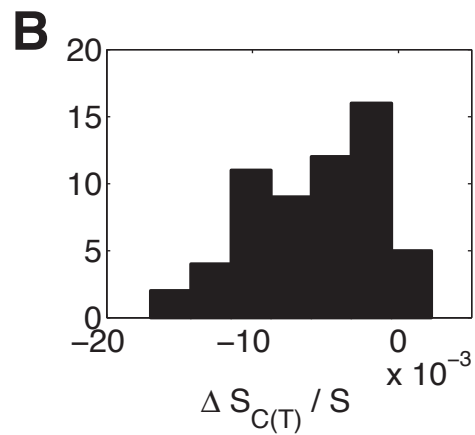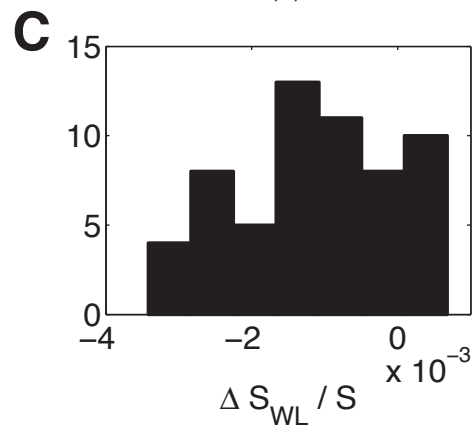

Supplement: Figure S2 — Precision of entropy estimates. (A) Entropy estimation using heat capacity integration (x-axis) from Eq (29) versus entropy estimation using the Wang-Landau sampling method (y-axis) [73]. Each plot symbol is one subnetwork of either N = 100 or N = 120 neurons (circles = pairwise models, crosses = K-pairwise models). The two sampling methods yield results that agree to within ∼1%. (B) Fractional difference between the heat capacity method and the entropy determined from the all-silent pattern. The histogram is over 30 networks at N = 100 and 30 at N = 120, for the K-pairwise model. (C) Fractional difference between the Wang-Landau sampling method and the entropy determined from the all-silent pattern. Same convention as in (B). (PDF) [file pcbi.1003408.s002.pdf]

**A**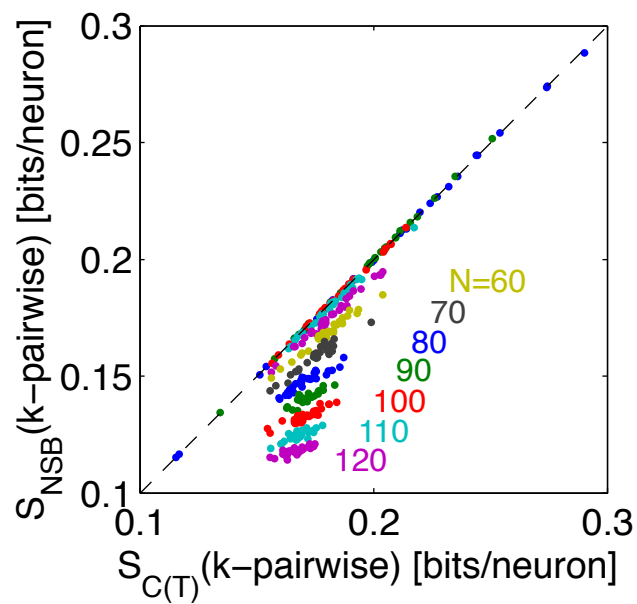**B**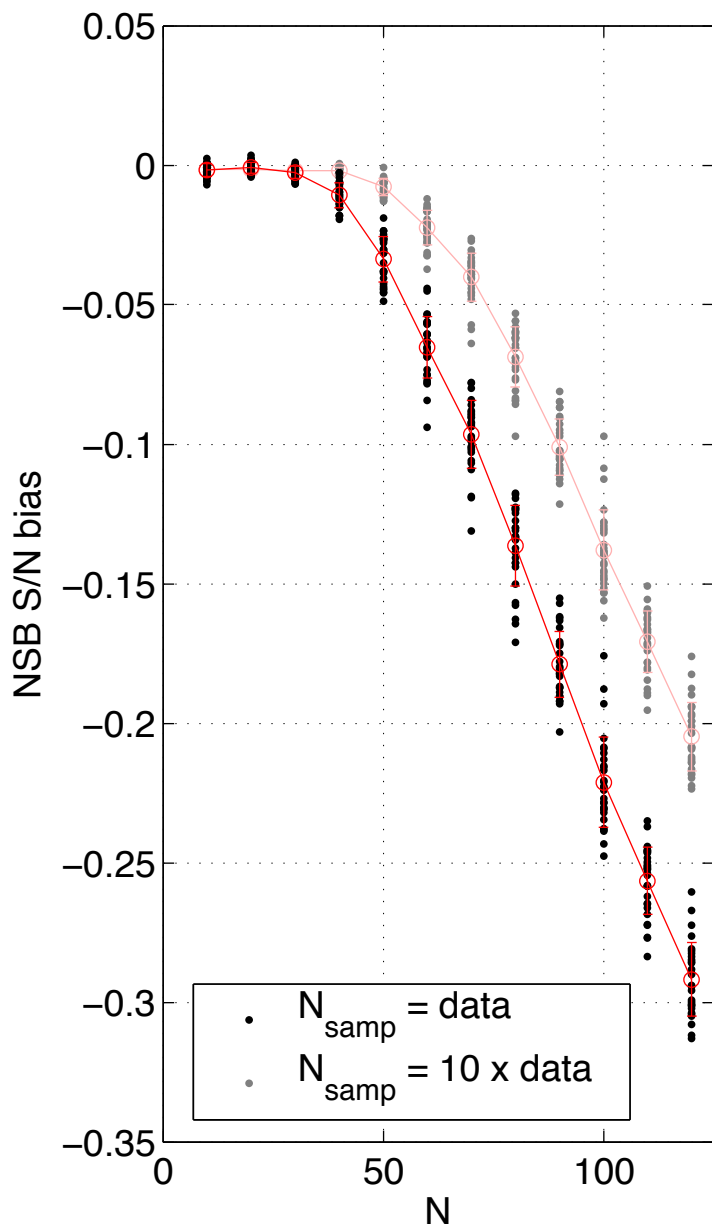**C**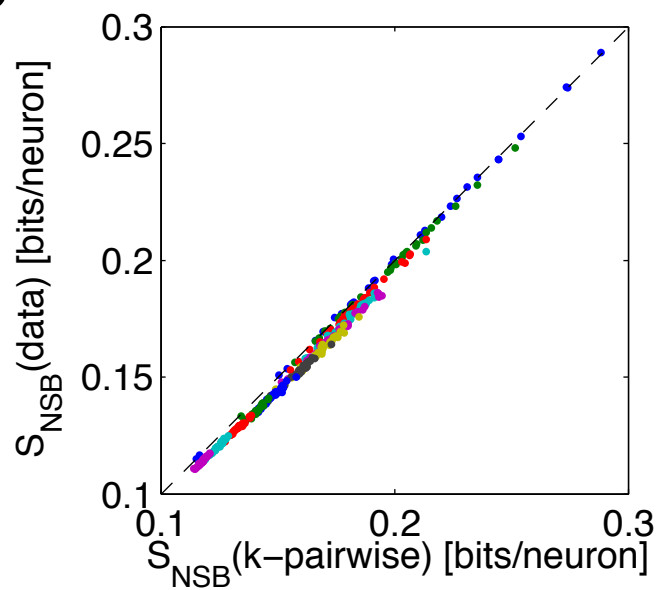

Supplement: Figure S3 — Sample-based entropy estimation. (A) The bias in entropy estimates computed directly from samples drawn from K-pairwise models. The NSB entropy estimate [71] in bits per neuron computed using samples from the model (same size as the experimental data set) on y-axis; the true entropy (using heat capacity integration) method on x-axis. Each dot represents one subnetwork of a particular size (N, different colors). For small networks () the bias is negligible, but estimation from samples significantly underestimates the entropy for larger networks. (B) The fractional bias of the estimator as a function of N (black dots = data from (A), gray dots = using 10 fold more samples). Red line shows the mean ± s.d. over 30 subnetworks at each size. (C) The NSB estimation of entropy from samples drawn from the model (x-axis) vs the samples from real experiment (y-axis); each dot is a subnetwork of a given size (color as in (A)). The data entropy estimate is slightly smaller than that of the model, as is expected for true entropy; for estimates from finite data this would only be expected if the biases on data vs MC samples were the same. (PDF) [file pcbi.1003408.s003.pdf]

**A** $J$  (perturbative)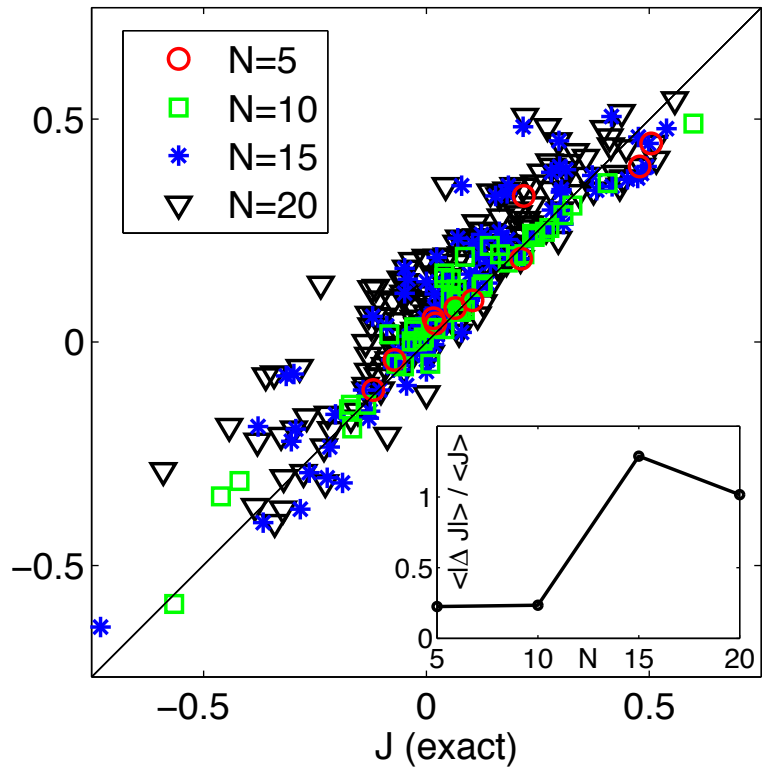**B** $D_{JS}(\text{model}; \text{data})$ 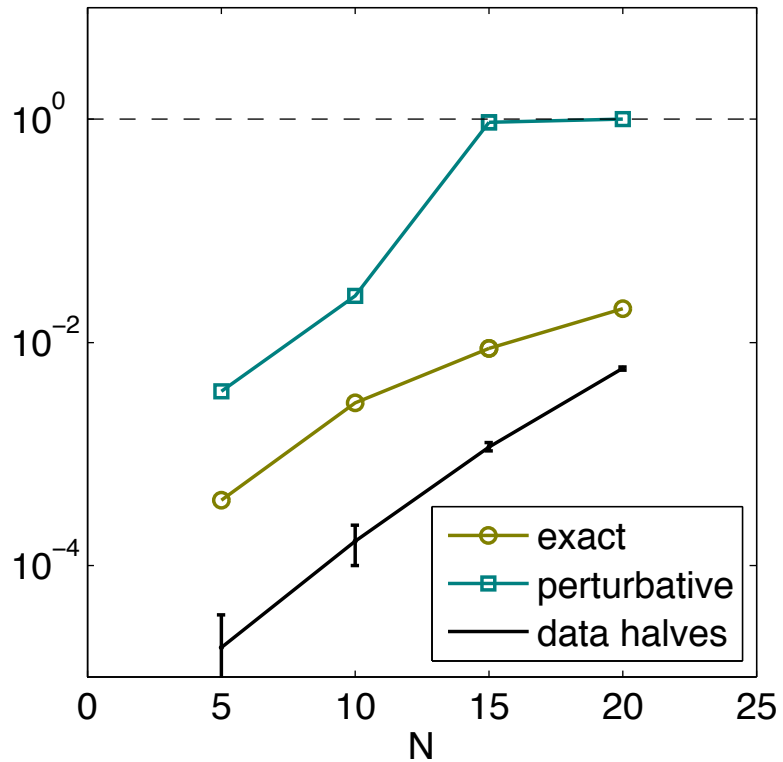

Supplement: Figure S4 — Perturbative vs exact solution for the pairwise maximum entropy models. (A) The comparison of couplings for a group of neurons, computed using the exact maximum entropy reconstruction algorithm, with the lowest order perturbation theory result, , where and [34], [113]. In the case of larger networks, the perturbative deviate more and more from equality (black line). Inset: the average absolute difference between the true and perturbative coupling, normalized by the average true coupling. (B) The exact pairwise model, Eq (19), can be compared to the distribution , sampled from data; the olive line (circles) shows the Jensen-Shannon divergence (corrected for finite sample size) between the two distributions, for four example networks of size . The turquoise line (squares) shows the same comparison in which the pairwise model parameters, , were calculated perturbatively. The black line shows the between two halves of the data for the four selected networks. (PDF) [file pcbi.1003408.s004.pdf]

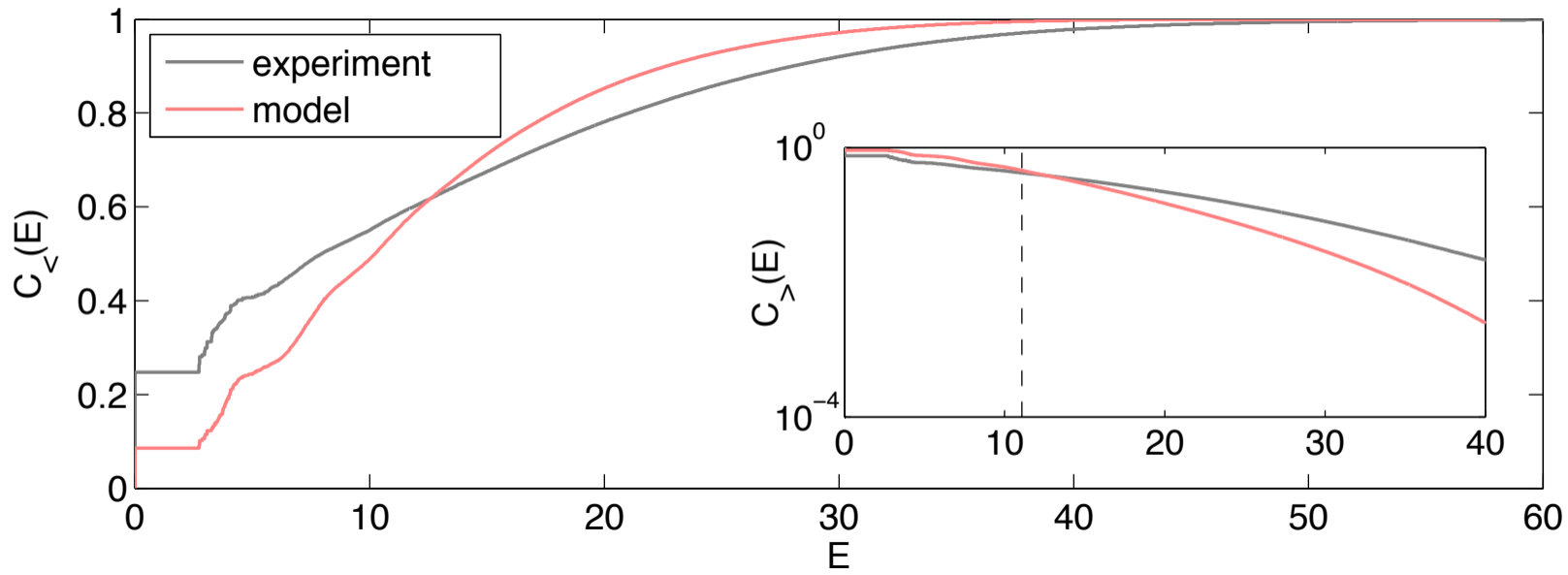

Supplement: Figure S5 — Predicted vs real distributions of energy, E, for the pairwise model. The cumulative distribution of energies, from Eq (22), for the patterns generated by the pairwise models (red) and the data (black), in a population of 120 neurons. Inset shows the high energy tails of the distribution, from Eq (24); dashed line denotes the energy that corresponds to the probability of seeing the pattern once in an experiment. This figure is analogous to Figure 8; the same group of neurons is used here. (PDF) [file pcbi.1003408.s005.pdf]

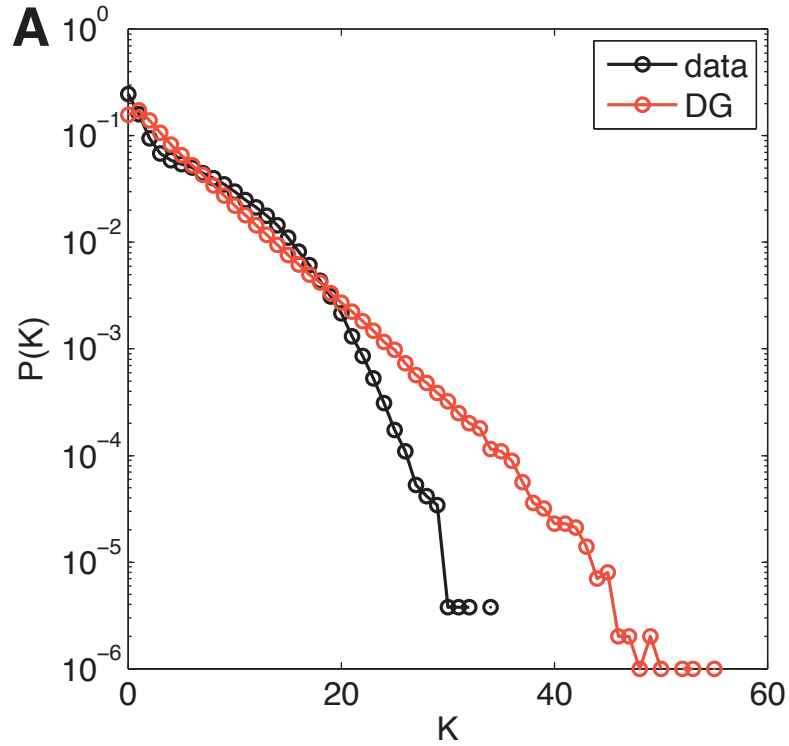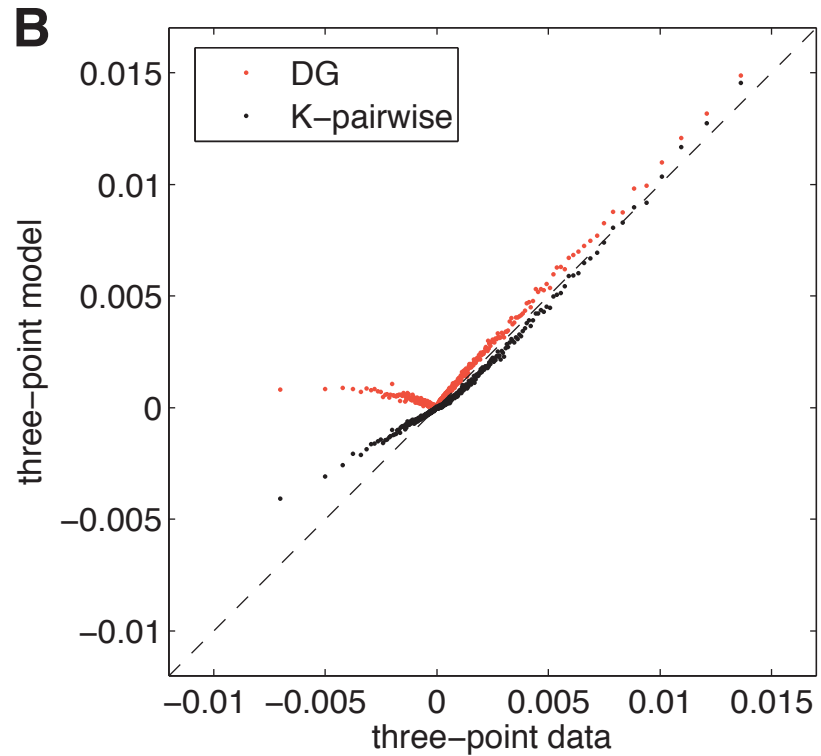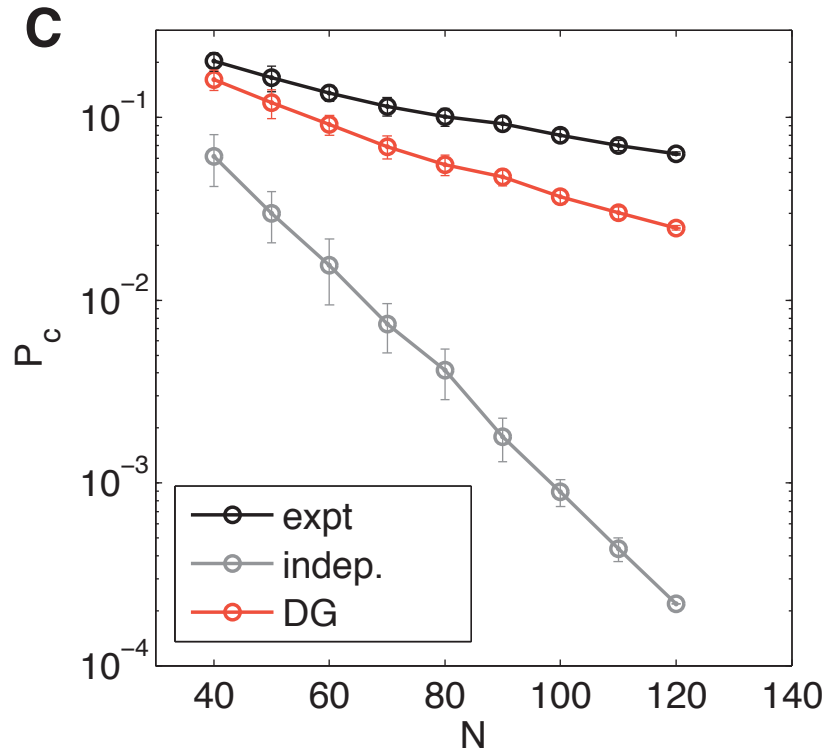

Supplement: Figure S6 — Dichotomized Gaussian model performance for a group of N = 120 neurons. (A) The distribution of synchronous spikes, P(K), in the data (black) and in the DG model fit to data (red). For this network, DG predicts ; the true value is . (B) The comparison of three-point correlations estimated from data (x-axis) and predicted by the two models (y-axis; red = DG, black = K-pairwise). As in Figure 7, three-point correlations are binned; shown are the means for the predictions in a given bin, error-bars are omitted for clarity. DG underperforms the K-pairwise model specifically for negative correlations. (C) The probability of coincidences, analogous to Figure 14, computed for the DG model (red) and compared to data (black); gray line is the independent model. (PDF) [file pcbi.1003408.s006.pdf]

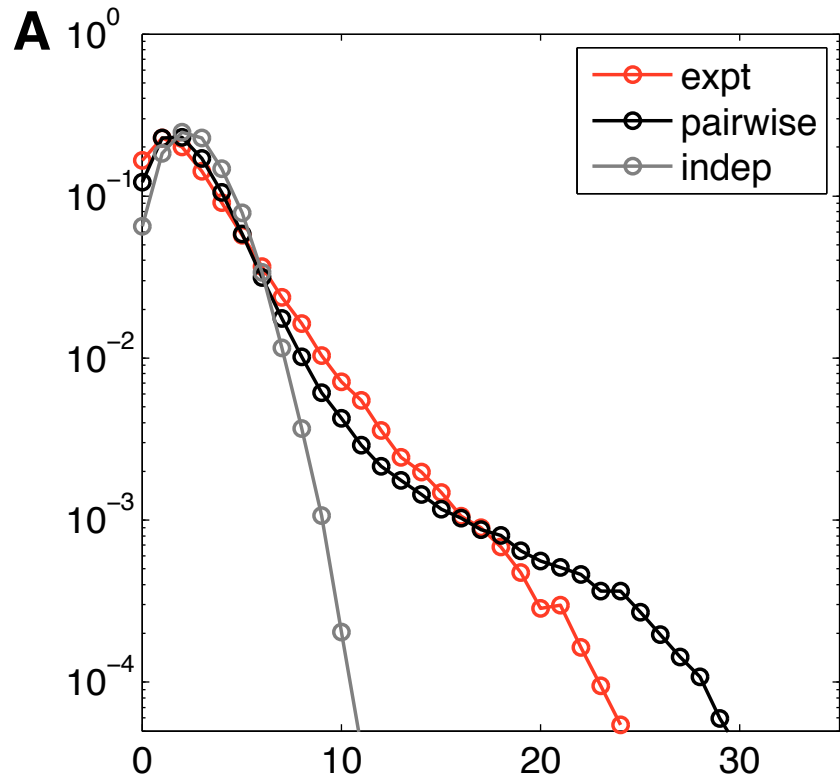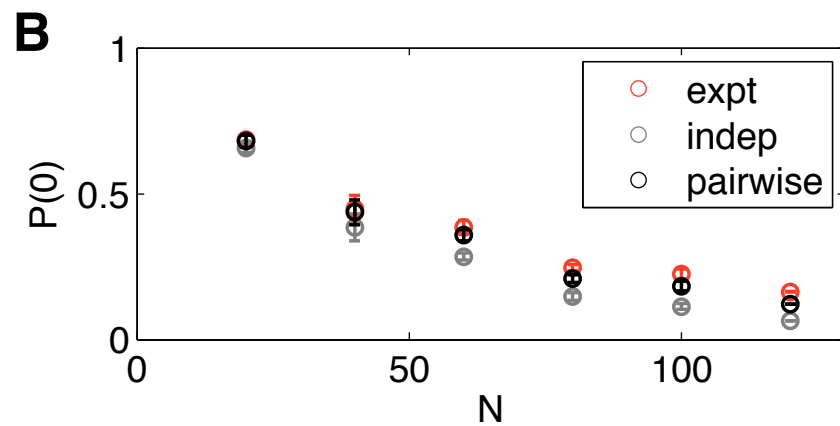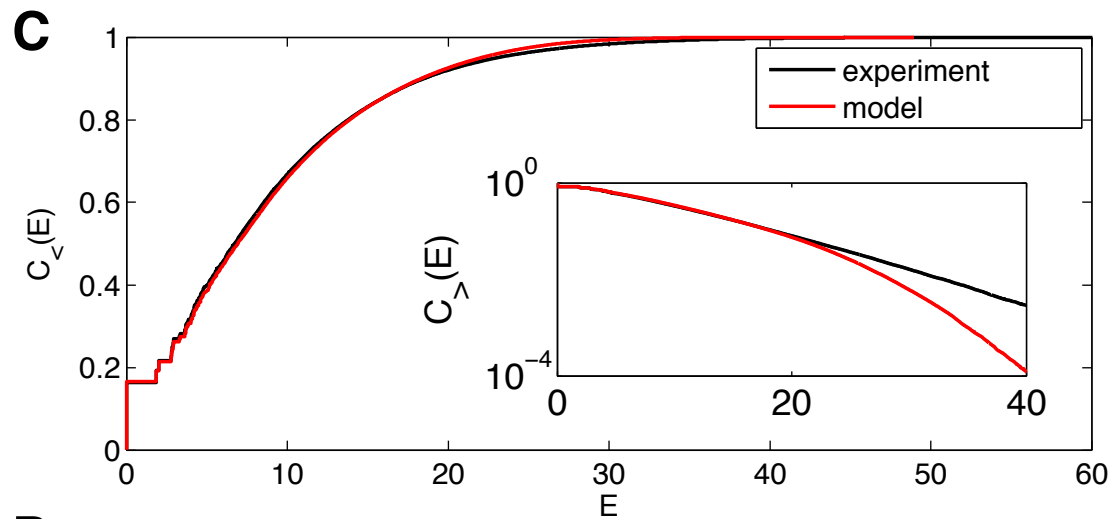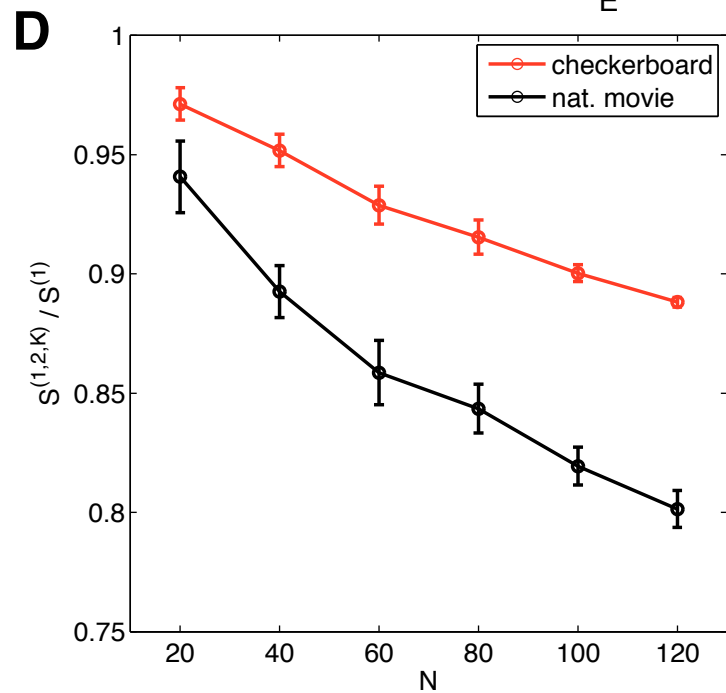

Supplement: Figure S7 — Maximum entropy models for the checkerboard stimulation. We stimulated a separate retina with a checkerboard stimulus. The square check size was 69 µm, smaller than the typical size of the ganglion cell receptive fields. Each check was randomly selected to be either black or white on each frame displayed at a rate of 30 Hz. The entire stimulus consisted of 69 repeats of 30 seconds each, and subgroups of up to 120 neurons were analyzed. (A) Distribution of synchrony, P(K), for a group of 120 neurons, in the data (red), as predicted by the pairwise model (black), and by the independent model (gray). (B) As the network of N neurons gets larger, the discrepancy in the prediction of the probability of silence, P(0), grows in a qualitatively similar way as under naturalistic stimulation. (C) K-pairwise models capture the distribution of energies very well even at N = 120 (cf. Figure 8 for an analogous plot for natural stimulation). (D) Under checkerboard stimulation, the distribution of codewords is less correlated than under the natural stimulation, as quantified by the ratio of the entropy to the independent entropy, shown as a function of subgroup size N. (PDF) [file pcbi.1003408.s007.pdf]
